# A randomized, single-blind, single-dose study evaluating the pharmacokinetic equivalence of proposed biosimilar ABP 980 and trastuzumab in healthy male subjects

**DOI:** 10.1007/s00280-017-3286-9

**Published:** 2017-03-24

**Authors:** Vladimir Hanes, Vincent Chow, Nan Zhang, Richard Markus

**Affiliations:** Biosimilars Development, Amgen Inc., One Amgen Center Drive, Thousand Oaks, CA 91320 USA

**Keywords:** Biosimilar, Pharmacokinetics, Trastuzumab, HER2, ABP 980, Immunogenicity

## Abstract

**Purpose:**

This study compared the pharmacokinetic (PK) profiles of the proposed biosimilar ABP 980 and trastuzumab in healthy males.

**Methods:**

In this single-blind study, 157 healthy males were randomized 1:1:1 to a single 6 mg/kg intravenous infusion of ABP 980, FDA-licensed trastuzumab [trastuzumab (US)], or EU-authorized trastuzumab [trastuzumab (EU)]. Primary endpoints were area under the serum concentration–time curve from time 0 to infinity (AUC_inf_) and maximum observed serum concentration (*C*_max_). To establish equivalence, the geometric mean ratio (GMR) and 90% confidence interval (CI) for *C*_max_ and AUC_inf_ had to be within the equivalence criteria of 0.80–1.25.

**Results:**

The GMRs and 90% CIs for *C*_max_ and AUC_inf_, respectively, were: 1.04 (0.99–1.08) and 1.06 (1.00–1.12) for ABP 980 versus trastuzumab (US); 0.99 (0.95–1.03) and 1.00 (0.95–1.06) for ABP 980 versus trastuzumab (EU); and 0.96 (0.92–1.00) and 0.95 (0.90–1.01) for trastuzumab (US) versus trastuzumab (EU). All comparisons were within the equivalence criteria of 0.80–1.25. Treatment-emergent adverse events (TEAEs) were reported in 84.0, 75.0, and 78.2 of subjects in the ABP 980, trastuzumab (US), and trastuzumab (EU) groups, respectively. There were no deaths or TEAEs leading to study discontinuation and no binding or neutralizing anti-drug anti-bodies were detected.

**Conclusions:**

This study demonstrated the PK similarity of ABP 980 to both trastuzumab (US) and trastuzumab (EU), and of trastuzumab (US) to trastuzumab (EU). No differences in safety and tolerability between treatments were noted; no subject tested positive for binding anti-bodies.

## Introduction

ABP 980 is being developed as a biosimilar to trastuzumab (Herceptin^®^). Herceptin^®^ is approved for use in the United States (US), the European Union (EU), Japan, and much of the rest of the world for the treatment of metastatic breast cancer, early breast cancer, and metastatic gastric cancer [1, 2], and is the standard of care for subjects with human epidermal growth factor receptor 2 (HER2)-overexpressing breast cancer [3–5]. Trastuzumab and ABP 980 are produced by recombinant DNA technology in Chinese hamster ovary (CHO) cells and both monoclonal anti-bodies (mAbs) bind with high affinity and specificity to the extracellular domain of the HER2 [6]. Binding of trastuzumab to HER2 blocks receptor activation and subsequent proliferation of HER2-overexpressing cells [7]. In addition to blocking HER2-mediated proliferation, binding of trastuzumab to HER2 elicits anti-body-dependent cell-mediated cytotoxicity (ADCC) by binding to antigens expressed on target cells, while the anti-body Fc domain engages Fc receptors (e.g., FcγRIIIa) on the surface of immune effector cells. This binding sequence leads to the activation of the effector cell, granule exocytosis, and target cell death [7].

There is increasing interest in biosimilars as alternatives to their respective reference products, since they can provide additional safe and efficacious treatment options for patients. A biosimilar is a biologic product that is similar to a reference product with respect to quality, safety, and efficacy [8, 9]. Biosimilars are likely to have differences in product quality attributes owing to differences in cellular expression systems, processes, and product purification processes but should not have clinically meaningful differences with respect to safety, purity, and potency. Guidelines describing non-clinical and clinical considerations for the development of biosimilars, including mAbs, were issued by the European Medicines Agency (EMA) in 2012 and 2014 [10, 11], and by the United States Department of Health and Human Services and its Food and Drug Administration (FDA) in 2014 and 2015 [12, 13].

Both FDA and EMA guidelines are similar in that each recommends a stepwise approach beginning with comprehensive analytical characterization to establish structural and functional similarity of the biosimilar with the reference or innovator product, followed by a pharmacokinetic (PK) study and a pharmacodynamic (PD) study, if a suitable PD marker is available, to establish equivalence. Finally, a comparative clinical study is conducted in a sensitive population to confirm similar efficacy, safety, and immunogenicity of the proposed biosimilar to the reference product.

ABP 980 is being developed for the same indications, dosages, and route of administration as trastuzumab. Previous studies have shown that ABP 980 has the same amino acid sequence and is analytically and functionally similar to trastuzumab [14]. This Phase I, single-dose, PK study in healthy male subjects was conducted to determine the PK equivalence of ABP 980 to US FDA-licensed trastuzumab and EU-authorized trastuzumab, hereafter referred to as trastuzumab (US) and trastuzumab (EU), respectively. The secondary objectives were to compare the safety, tolerability, and immunogenicity of ABP 980, trastuzumab (US), and trastuzumab (EU). Finally, to establish a scientific bridge between reference products sourced from the US and the EU, the PK equivalence between trastuzumab (US) and trastuzumab (EU) was evaluated.

## Materials and methods

This study was conducted in accordance with the provisions of the Declaration of Helsinki and in accordance with the ICH E6 Guidelines on Good Clinical Practice. All subjects signed an Institutional Review Board (IRB)/Independent Ethics Committee (IEC)-approved informed consent form before any study-specific procedures were performed.

### Investigational product

ABP 980 was sourced from Amgen, Inc. (Thousand Oaks, CA, USA). Trastuzumab (US) was sourced from Genentech, Inc. (South San Francisco, CA, USA), and trastuzumab (EU) was sourced from Roche Pharma AG (Grenzach-Wyhlen, Germany). ABP 980 and trastuzumab (US) were supplied in vials containing 440 mg of investigational product, and trastuzumab (EU) was supplied in vials containing 150 mg of investigational product. All investigational products were reconstituted to 21 mg/mL. The protein content of each of the drug lots obtained by the clinical sites was evaluated by Amgen, Inc. No adjustment to dosage was necessary as the differences in protein content between formulations were determined to be less than 5%.

### Study design and subject population

**Fig. 1:**
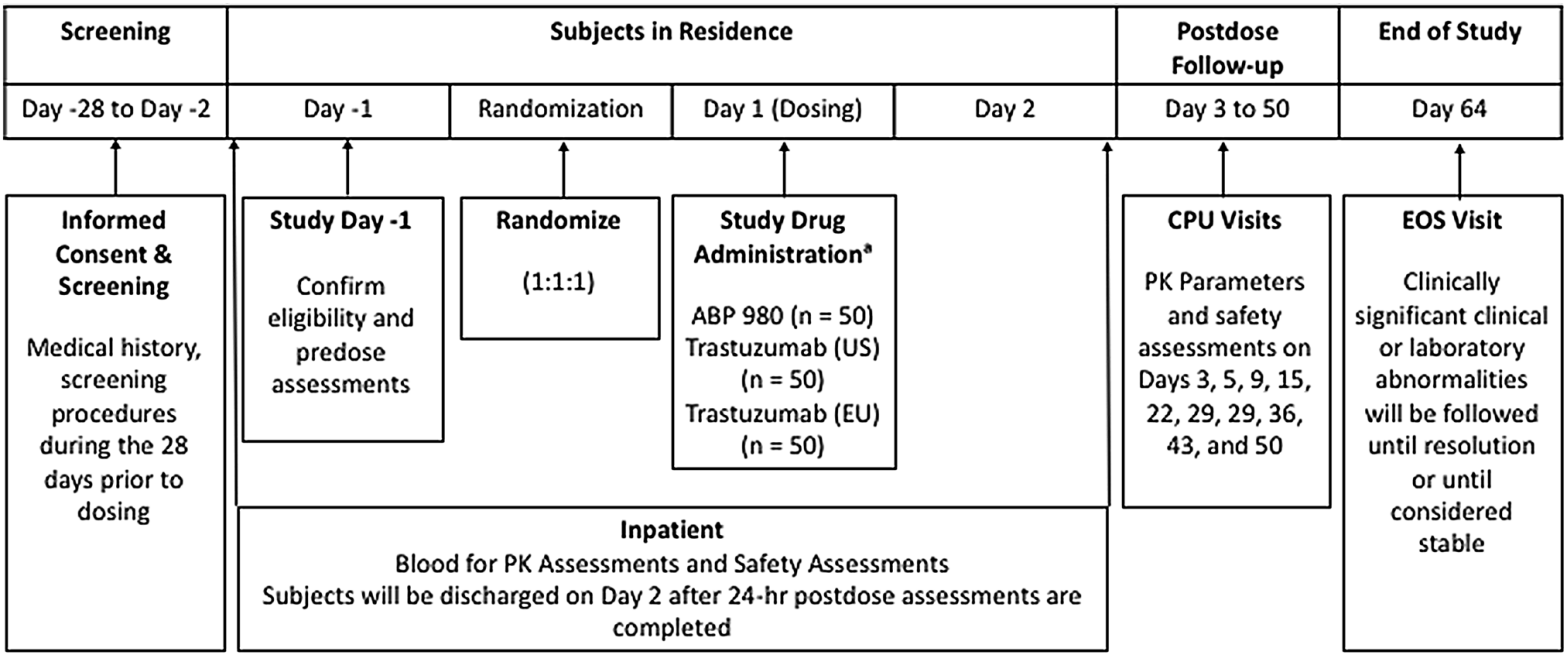
Study design. *CPU* clinical pharmacology unit, *EOS* end of study, *EU* European Union, *FDA* Food and Drug Administration, *IV* intravenous, *PK* pharmacokinetic, *US* United States. ^a^Planned IV dose: ABP 980 6 mg/kg, 440 mg vial; FDA-licensed trastuzumab 6 mg/kg, 440 mg vial; or EU-authorized trastuzumab 6 mg/kg, 150 mg vial

This randomized, single-blind, single-dose, three-arm, parallel-group study in healthy adult males was conducted at a single center (Fig. 1). A total of 150 healthy male subjects were planned to be enrolled in the study.

Subjects were randomized in a ratio of 1:1:1, stratified by ethnicity (Japanese versus non-Japanese), to receive a single intravenous (IV) infusion of ABP 980 6 mg/kg, trastuzumab (US) 6 mg/kg, or trastuzumab (EU) 6 mg/kg over 90 min. Subjects, but not healthcare professionals administering the investigational product, were blinded to which treatment they were receiving.

Eligible participants included healthy adult men ≥18–≤45 years of age. Inclusion criteria comprised normal or clinically acceptable physical examination, clinical laboratory test values, vital signs, echocardiogram, and electrocardiogram (ECGs) at screening, body mass index (BMI) ≥18.0 and ≤30.0 kg/m^2^ for non-Japanese subjects, and BMI ≥18.0 and ≤25.0 kg/m^2^ for Japanese subjects. Physical examination, ECGs, and vital signs were repeated on Day-1.

Exclusion criteria included men of reproductive potential unwilling to practice a highly effective method of birth control, or refrain from donating sperm, for the duration of the study and for 5 months after treatment and men with pregnant partners. Additional exclusion criteria included a history or evidence of a clinically significant disorder, condition, or disease that could pose a risk to subject safety or interfere with the study; history or presence of conditions known to interfere with the distribution, metabolism, or excretion of drugs; history of surgery or major trauma within 12 weeks of screening, or surgery planned during the study; prior exposure to trastuzumab or related compounds; known or suspected sensitivity to products derived from mammalian cell lines; and subjects who were receiving or had received any investigational drug, device, or medication (prescription or over-the-counter) within 30 days or 5 half-lives (whichever was longer) of receiving study medication.

Subjects were screened and informed consent was obtained within 28 days of treatment administration. Day-1 assessment included medical history, physical examination, vital signs, and body weight. Body weight on Day-1 was used to calculate dose. Day 1 pre-dose procedures and assessments included vital signs and collection of samples for baseline assessment of hematology, chemistry, urinalysis, and ADAs.

Subjects remained in the study center or clinical pharmacology unit (CPU) for at least 24 h after dosing for safety and PK assessments. They were discharged on Day 2 after the 24-h assessments were completed. Subjects returned to the CPU on Days 3, 5, 9, 15, 22, 29, 36, 43, and 50, and Day 64 [end-of-study (EOS) visit] for safety evaluations and PK assessments. Vital signs and laboratory measurement were taken on Days 1 and 64 (EOS).

### Sampling

Blood samples for serum ABP 980 or trastuzumab concentration determination were collected pre-dose, at 0.75 (mid-infusion), 1.5 (end of infusion), 2, 3, 4, 5, 6, 8, and 24 h after the start of dosing, and at each return visit to the CPU. Blood sampling for PK analysis during each return visit to the CPU, including the EOS visit, occurred per the scheduled timepoint.

A validated electrochemiluminescence (ECL) assay was used to quantify serum concentrations of ABP 980 and trastuzumab using a mouse anti-trastuzumab monoclonal anti-body (mAb) to capture the investigational product. After capturing ABP 980 or trastuzumab to the immobilized anti-body, unbound materials were removed, followed by the addition of ruthenium labeled mouse anti-trastuzumab mAb to detect the captured ABP 980 or trastuzumab. A tripropylamine buffer was added to enhance the electrochemiluminescent signals. The ECL counts were directly proportional to the amount of ABP 980 or trastuzumab bound by the capture reagent. Conversion of ECL counts to concentrations was performed using the Gen5™ Secure Software v1.08.

Safety and tolerability were reviewed by the medical monitor on an ongoing basis. Adverse events (AEs) were monitored throughout the study. All AEs and serious AEs (SAEs) were reported. Adverse events were coded using MedDRA Version 17.0.

### Anti-drug anti-bodies assay

Binding and neutralizing ADAs were detected with a two-tiered approach that included a screening assay and a confirmatory assay. Sampling for ADAs occurred on Day 1 pre-dose and at the EOS visit. A validated immunoassay was used to detect anti-bodies capable of binding ABP 980, trastuzumab (US), or trastuzumab (EU). All samples positive for binding ADAs were assessed for neutralizing anti-bodies capable of binding to ABP 980, trastuzumab (US), or trastuzumab (EU) using a target binding assay.

### Study endpoints

The primary endpoints were AUC_inf_ and *C*_max_ for ABP 980, trastuzumab (US), and trastuzumab (EU). Secondary endpoints included terminal elimination half-life (*t*_½_); the time at which *C*_max_ was observed (*t*_max_); AUC from time 0 to the last quantifiable concentration (AUC_last_); and last measureable serum concentration (*C*_last_) for ABP 980 and trastuzumab. Although AUC_last_ was not defined as a primary endpoint, to fully assess exposure to the investigational product, AUC_last_ was also statistically evaluated. Secondary safety and immunogenicity endpoints included the incidence of treatment-emergent AEs (TEAEs), SAEs, and ADAs.

Pharmacokinetic equivalence was established if the 90% confidence intervals (CIs) for the ratio of least square geometric means (GMs) of primary PK parameters comparing ABP 980 versus trastuzumab (US), ABP 980 versus trastuzumab (EU), and trastuzumab (US) versus trastuzumab (EU) fell within the standard equivalence criteria of 0.80 and 1.25.

### Statistical analysis

Serum trastuzumab and ABP 980 concentrations were listed and summarized descriptively by treatment and timepoint using the PK concentration population including all subjects who were randomized and received any amount of investigational product and had at least one reported serum concentration of ABP 980 or trastuzumab. Mean trastuzumab and ABP 980 serum concentration–time data by treatment were presented graphically on semi-logarithmic and linear scales.

PK parameters were calculated using non-compartmental techniques (WinNonlin^®^ Professional Network Edition, Version 6.3, Pharsight Corp, St. Louis, MO) for all subjects. Prior to statistical modeling, PK parameters were log_e_-transformed. Point estimates and 90% CIs for the mean difference in logarithmic PK parameters were estimated using an analysis of variance (ANOVA) model adjusted for treatment and ethnicity using the PK parameter population including all subjects with an evaluable ABP 980 or trastuzumab serum concentration–time profile. The point estimates and 90% CIs for GM ratios (GMRs) were then calculated by transforming back to the original scale and PK similarity was established if the 90% CIs fell within the standard equivalence criteria of 0.80 and 1.25.

Safety analysis included descriptive summaries of AEs and the incidence of ADAs, using the safety population defined as all subjects who received any amount of investigational product.

## Results

### Subject disposition and characteristics

A total of 157 subjects were randomized to treatment with ABP 980 (*n* = 50), trastuzumab (US) (*n* = 52), and trastuzumab (EU) (*n* = 55); 148 (94.3%) subjects completed the study; 9 (5.7%) subjects [3 subjects in the trastuzumab (US) group and 6 subjects in the trastuzumab (EU) group] discontinued from the study prematurely. One subject in the trastuzumab (EU) group prematurely discontinued infusion owing to an infusion reaction. This subject received a total volume infused of approximately 4.8 mg/kg and was excluded from the PK parameter and per protocol PK analysis. No subject had a medical or surgical history that prohibited them from participation in the study. Subject demographics are summarized in Table 1. Age, height, weight, and BMI were similar across treatment groups. The majority of subjects (62.4%) in the study were white.

**Table 1:** Demographic data and baseline characteristics

	ABP 980 (*n* = 50)	Trastuzumab (US) (*n* = 52)	Trastuzumab (EU) (*n* = 55)
Mean age, years (SD)	25.8 (5.8)	25.4 (5.6)	25.6 (4.6)
Mean weight, kg (SD)	76.4 (11.1)	73.6 (10.8)	74.7 (11.8)
Mean height, cm (SD)	176.5 (6.25)	177.2 (7.37)	177.6 (8.01)
Mean BMI, kg/m^2^ (SD)	24.4 (2.8)	23.3 (2.5)	23.6 (3.4)
Race, *n* (%)			
Asian (1st generation Japanese)	10 (20.0)	10 (19.2)	11 (20.0)
Asian (other)	8 (16.0)	6 (11.5)	5 (9.1)
Black or African-American	3 (6.0)	0	0
White	28 (56.0)	34 (65.4)	36 (65.5)
Other	1 (2.0)	2 (3.8)	3 (5.5)

*BMI* body mass index, *SD* standard deviation

### Pharmacokinetic profiles

**Fig. 2:**
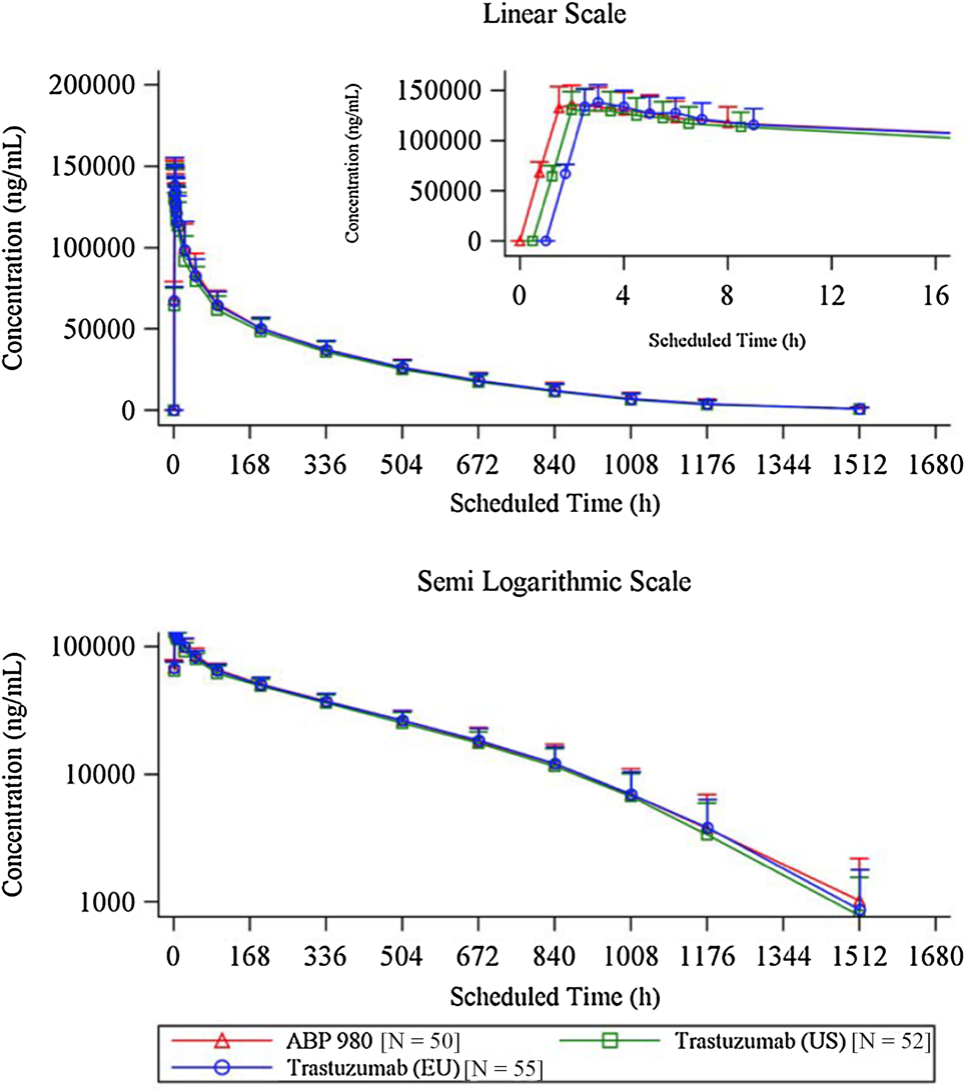
Mean serum concentration–time profiles (PK concentration population) for ABP 980, trastuzumab (US), and trastuzumab (EU); linear scale (*top*) and expanded (0–16 h) scale (*top inset*); semi-logarithmic scale (*bottom*). * Error bars* SD

The mean serum concentration–time profiles were similar between treatments following a single 6 mg/kg IV infusion of ABP 980, trastuzumab (US), or trastuzumab (EU) over the entire course of the study (Fig. 2). Peak concentrations were observed approximately 1.5–5 h after the start of the infusion with similar *C*_max_ and *t*_max_ in all treatment groups.

Descriptive summary of PK parameters for ABP 980, trastuzumab (US), and trastuzumab (EU) is presented in Table 2. A total of 12 subjects [4 subjects in the trastuzumab (US) group and 8 subjects in the trastuzumab (EU) group] either terminated the study early or had multiple missing samples. These subjects were included in the PK Parameter Population, as *C*_max_ and *t*_max_ were considered to have been sufficiently characterized before the early termination or missing samples; however, AUC_inf_ was considered non-evaluable in these subjects and was, therefore, excluded from analysis. The GMs of PK parameters were similar across treatment groups following a single IV infusion of ABP 980, trastuzumab (US), and trastuzumab (EU) (Table 2). The GMs of *C*_max_ and AUC_inf_ were 139.9 μg/mL and 35,224 μg h/mL for ABP 980; 134.6 μg/mL and 33,342 μg h/mL for trastuzumab (US); and 140.5 μg/mL and 35,123 μg h/mL for trastuzumab (EU). The terminal t_½_ was estimated to be 6–7 days. For all subjects in each treatment arm, AUC_last_ accounted for ≥90% of the total AUC, confirming the adequacy of the duration of PK sampling across the three treatments.

**Table 2:** Summary of pharmacokinetic parameters

Parameter	ABP 980	Trastuzumab (US)	Trastuzumab (EU)
*C*_max_ (μg/mL), GM (*n*) (GeoCV%)	139.9 (50) (13)	134.6 (52) (13)	140.5 (54) (12)
*C*_last_ (μg/mL), GM (*n*) (GeoCV%)	0.717 (50) (97)	0.574 (48) (91)	0.630 (46) (97)
AUC_last_, (μg h/mL), GM (*n*) (GeoCV%)	34,945 (50) (17)	33,160 (48) (17)	34,896 (46) (17)
AUC_inf_, (μg h/mL), GM (*n*) (GeoCV%)	35,224 (50) (18)	33,342 (48) (17)	35,123 (46) (18)
*t*_max_ (h), median (range) (*n*)	2.0 (1.52–5.00) (50)	2.0 (1.53–5.00) (52)	2.0 (1.55–24.00) (54)
*t*_½_ (h), mean (SD) (*n*)	169.4 (40.82) (50)	154.0 (27.97) (48)	154.8 (39.78) (46)

*GeoCV%* geometric mean coefficient of variation, *AUC* area under the serum concentration–time curve, *AUC*_*inf*_ AUC from time 0 extrapolated to infinity, *AUC*_*last*_ AUC from time 0 to the last quantifiable concentration, *CI* confidence interval, *C*_*max*_ maximum serum concentration, *LS* least squares, *t*_*max*_ time at which the maximum serum concentration was observed, *t½* terminal elimination half-life, *Max* maximum, *Min* minimum, *SD* standard deviation, *GM* geometric mean, *n* number of non-missing observations

The results of the statistical assessment of PK similarity for the overall population are shown in Table 3. The GMRs and 90% CIs for *C*_max_ and AUC_inf_, respectively, were: 1.04 (0.99–1.08) and 1.06 (1.00–1.12) for ABP 980 versus trastuzumab (US); 0.99 (0.95–1.03) and 1.00 (0.95–1.06) for ABP 980 versus trastuzumab (EU); and 0.96 (0.92–1.00) and 0.95 (0.90–1.01) for trastuzumab (US) versus trastuzumab (EU). All comparisons were within the equivalence criteria of 0.80–1.25.

**Table 3:** Statistical assessment of pharmacokinetic parameters

	PK parameters		
	*C*_max_ (μg/mL) Adjusted LS GM (*n*)	AUC_inf_ (μg h/mL) Adjusted LS GM (*n*)	AUC_last_ (μg h/mL) Adjusted LS GM (*n*)
ABP 980	135.9 (50)	34061.4 (50)	33811.7 (50)
Trastuzumab (US)	131.2 (52)	32271.7 (48)	32113.6 (48)
Trastuzumab (EU)	136.8 (54)	33947.0 (46)	33748.2 (46)
Statistical analysis: ratio and 90% CI of adjusted least square geometric means			
ABP 980 versus trastuzumab (US)	1.04 (0.9948–1.0787)	1.06 (0.9974–1.1169)	1.05 (0.9967–1.1122)
ABP 980 versus trastuzumab (EU)	0.99 (0.9540–1.0338)	1.00 (0.9476–1.0624)	1.00 (0.9479–1.0589)
Trastuzumab (US) vs trastuzumab (EU)	0.96 (0.9213–0.9975)	0.95 (0.8973–1.0072)	0.95 (0.8998–1.0063)

Statistical model includes treatment and ethnicity as fixed effects

*GM* geometric means, *LS* least squares, *n* number of non-missing observations

### Safety results

Overall, the incidence of TEAEs was similar across treatment groups. Treatment-emergent AEs were reported in 42 (84.0%) subjects in the ABP 980 group, 39 (75.0%) subjects in the trastuzumab (US) group, and 43 (78.2%) subjects in the trastuzumab (EU) group. The majority of TEAEs were mild to moderate in severity. Treatment-related AEs (i.e., TEAEs assessed as possibly or probably related to study drug) were reported in 33 (66.0%), 33 (63.5%), and 39 (70.9%) subjects in the ABP 980, trastuzumab (US), and trastuzumab (EU) groups, respectively.

There were no deaths or life-threatening TEAEs or TEAEs leading to study discontinuation. Two subjects reported TEAEs rated as severe; one subject in the trastuzumab (EU) group had an SAE of infusion-related reaction that was considered probably related to study drug; and one subject in the trastuzumab (US) group had multiple SAEs resulting from a motor bike accident, including pulmonary embolism secondary to deep vein thrombosis, not related to study medication. None of the SAEs resulted in premature study discontinuation.

The most frequently reported TEAEs (reported in >5% of subjects in any treatment group) were headache, upper respiratory tract infection, chills, pyrexia, myalgia, nausea, epistaxis, arthralgia, and lethargy (Table 4). No new or unexpected safety signals were identified, and the safety profiles were consistent with what is known for trastuzumab.

**Table 4:** Summary of treatment-emergent adverse events (safety population)

MedDRA preferred term	ABP 980 (*n* = 50)		Trastuzumab (US) (*n* = 52)		Trastuzumab (EU) (*n* = 55)	
	*n* (%)	No. of events	*n* (%)	No. of events	*n* (%)	No. of events
Headache	16 (32.0)	21	19 (36.5)	26	24 (43.6)	30
Upper respiratory tract infection	21 (42.0)	25	18 (34.6)	20	20 (36.4)	22
Chills	3 (6.0)	3	6 (11.5)	7	7 (12.7)	8
Pyrexia	3 (6.0)	3	5 (9.6)	5	8 (14.5)	8
Myalgia	8 (16.0)	8	3 (5.8)	3	1 (1.8)	1
Nausea	2 (4.0)	2	5 (9.6)	5	4 (7.3)	4
Epistaxis	3 (6.0)	4	3 (5.8)	3	3 (5.5)	3
Arthralgia	5 (10.0)	5	1 (1.9)	1	1 (1.8)	1
Lethargy	3 (6.0)	3	2 (3.8)	2	0	0

Adverse events were coded using MedDRA Version 17.0

A TEAE is defined as an AE that was not present prior to treatment with investigational product, but appeared following treatment or was present at treatment initiation but worsened during treatment. Subjects with multiple events in the same category were counted only once in that category; subjects with events in more than 1 category were counted once in each of those categories

### Anti-drug anti-bodies

Immunogenicity was assessed at baseline just prior to dosing and at the EOS visit (Day 64). For all subjects, the concentrations of ABP 980 or trastuzumab at the end of the PK sampling period were well below the drug tolerance levels of 20 and 100 μg/mL in the presence of 100 and 500 ng/mL ADA, respectively, indicating that ADA detection was not influenced by circulating drug levels. There were no pre-existing binding ADAs detected in baseline samples and no subjects had developed binding or neutralizing ADAs at the end of the study.

## Discussion

This study was conducted to determine the PK similarity of the proposed biosimilar ABP 980 to trastuzumab. Study enrollment was restricted to healthy male subjects, because healthy subjects should provide the most homogenous population for sensitive comparisons of the PK of ABP 980 and trastuzumab. The dose and sampling schedule chosen in this study were based on a previous study of trastuzumab [15]. A dose of 6 mg/kg provided sufficient exposure to study medication for an accurate evaluation of PK in healthy subjects within the dose range with linear kinetics and is, therefore, appropriate to detect potential PK differences between ABP 980, trastuzumab (US), and trastuzumab (EU). A dose of 6 mg/kg is also consistent with the prescribing instructions for Herceptin^®^ [1, 2]. Based on *t*_½_ of 6–6.8 days in healthy males in a prior pilot study for ABP 980 and trastuzumab (EU), 64 days of PK sampling post-infusion was considered sufficient to fully characterize the ABP 980 or trastuzumab PK profiles in healthy subjects.

This study was conducted to meet FDA and EMA guidelines for the development and approval of biosimilar agents [10–13]. Both agencies recommend a stepwise developmental approach designed to determine the similarity of the proposed biosimilar to the reference product with respect to physicochemical and functional characteristics, PK profile, and clinical efficacy, safety, and tolerability. The analytical and functional similarity of ABP 980 to trastuzumab sourced from the USA and EU has been extensively studied, and the results have been reported previously [14]. ABP 980 was shown to be similar to trastuzumab (US) and trastuzumab (EU) with respect to primary and higher order structure, HER2 binding affinity, inhibition of proliferation, and in vitro anti-body-dependent cell-mediated cytotoxicity. The results of this study further support that ABP 980 is similar to trastuzumab by demonstrating equivalence of ABP 980 to both trastuzumab (US) and trastuzumab (EU) with respect to PK profile.

A single 6 mg/kg IV infusion of ABP 980 in healthy male subjects resulted in a similar PK profile to both trastuzumab (US) and trastuzumab (EU) with respect to the primary PK parameters of AUC_inf_ and *C*_max_. For both PK parameters, the 90% CIs were contained within the prespecified standard equivalence margin of 0.8–1.25. The safety and tolerability of ABP 980, trastuzumab (US), and trastuzumab (EU) also were comparable and consistent with what is known for trastuzumab. No new or unexpected safety signals were noted.

The study design used here also allowed a direct comparison between trastuzumab (US) and trastuzumab (EU). Direct comparison between reference products sourced from different regions also has important regulatory and clinical implications. Strong evidence for similarity between ABP 980 and trastuzumab, based on comprehensive analytical and functional assessment, is necessary to support clinical studies and ultimately extrapolation to all approved trastuzumab indications [16, 17]. The FDA and EMA have each developed guidelines that allow the use of foreign-sourced reference products in comparative clinical trials provided that there is sufficient scientific evidence demonstrating similarity between the foreign-sourced and locally-sourced reference products. In this three-arm study, the 90% CIs for AUC_inf_ and *C*_max_ were within the equivalence margin of 0.8 to 1.25 for trastuzumab (US) versus trastuzumab (EU), hence established PK bioequivalence between USA-sourced trastuzumab and EU-sourced trastuzumab, which is a required component to establish a scientific bridge between USA-sourced and EU-sourced trastuzumab, thus allowing use of one reference product in comparative Phase III clinical trials. Safety and tolerability also were comparable between trastuzumab (US) versus trastuzumab (EU).

As with all biologic agents, the risk for developing binding or neutralizing ADAs must be carefully assessed. In this study, immunogenicity was evaluated by assaying blood samples for the presence of binding or neutralizing ABAs on Day 1 before infusion and at the end-of-study visit. No subject in any treatment arm developed ADAs. This finding is consistent with previous studies demonstrating a low incidence of ADAs with trastuzumab [15, 18].

In conclusion, in this Phase I study, there were no differences between ABP 980, trastuzumab (US), and trastuzumab (EU) with respect to PK profile, safety, and tolerability after a single IV infusion. No subject tested positive for binding ADAs. In addition to the results of structural and functional characterization, these results provide further support that the proposed biosimilar ABP 980 is highly similar to FDA-licensed and EU-authorized trastuzumab reference products.

## References

[1] (2016) Herceptin^®^ (trastuzumab) prescribing information. Genentech, Inc. http://www.accessdata.fda.gov/drugsatfda_docs/label/2010/103792s5250lbl.pdf. Accessed 21 March 2017

[2] (2014) Herceptin^®^ (trastuzumab) summary of product characteristics. Roche Registration Limited. http://www.ema.europa.eu/docs/en_GB/document_library/EPAR_-_Product_Information/human/000278/WC500074922.pdf. Accessed 21 March 2017

[3] Aebi S, Davidson T, Gruber G, Castiglione M, ESMO Guidelines Working Group (2010). Primary breast cancer: ESMO Clinical Practice Guidelines for diagnosis, treatment and follow-up. Ann Oncol.

[4] Cardoso F, Senkus-Konefka E, Fallowfield L, Costa A, Castiglione M, ESMO Guidelines Working Group (2010). Locally recurrent or metastatic breast cancer; ESMO Clinical Practice Guidelines for diagnosis, treatment and follow-up. Ann Oncol.

[5] National Comprehensive Cancer Network (NCCN) (2017) Clinical practice guidelines in oncology: breast cancer. Version 2.2016. https://www.nccn.org/professionals/physician_gls/pdf/breast.pdf. Accessed 16 Feb 2017

[6] Arnould L, Gelly M, Penault-Llorca F (2006). Trastuzumab-based treatment of HER2-positive breast cancer: an antibody-dependent cellular cytotoxicity mechanism?. Br J Cancer.

[7] Baselga J, Albanell J, Molina MA, Arribas J (2001). Mechanism of action of trastuzumab and scientific update. Semin Oncol.

[8] European Medicines Agency (2015) Guideline on similar biological medicinal products containing biotechnology-derived proteins as active substance: non-clinical and clinical issues. http://www.ema.europa.eu/docs/en_GB/document_library/Scientific_guideline/2015/01/WC500180219.pdf. Accessed 25 Aug 2016

[9] Christi L. FDA’s overview of the regulatory guidance for the development and approval of biosimilar products in the US. http://www.fda.gov/downloads/drugs/developmentapprovalprocess/howdrugsaredevelopedandapproved/approvalapplications/therapeuticbiologicapplications/biosimilars/ucm428732.pdf. Accessed 25 Aug 2016

[10] European Medicines Agency, Committee for Medicinal Products for Human Use (2012) Guideline on similar biological medicinal products containing monoclonal antibodies—non-clinical and clinical issues. http://www.ema.europa.eu/docs/en_GB/document_library/Scientific_guideline/2012/06/WC500128686.pdf. Accessed 7 Jul 2016

[11] Committee for Medicinal Products for Human Use (2014) Guideline on similar biological medicinal products. London, UK. Report No.: CHMP/437/04 rev 1

[12] US Food and Drug Administration (2014). Clinical pharmacology data to support a demonstration of biosimilarity to a reference product.

[13] US European Medicines Agency (2015) Scientific considerations in demonstrating biosimilarity to a reference product. Guidance for industry. http://www.fda.gov/downloads/Drugs/GuidanceComplianceRegulatoryInformation/Guidances/UCM291128.pdf. Accessed 7 Jul 2016

[14] Hutterer K, McBride H, Polozova A, Kuhns S, Liu J (2017) Analytical and functional similarity of proposed Amgen biosimilar ABP 980 to trastuzumab. 21st symposium on the interface of regulatory and analytical sciences for biotechnology health products, January 24–26, 2017; Washington, DC. Poster number P-207-TH

[15] Wynne C, Harvey V, Schwabe C (2013). Comparison of subcutaneous and intravenous administration of trastuzumab: a phase I/Ib trial in healthy male volunteers and patients with HER2-positive breast cancer. J Clin Pharmacol.

[16] Weise M, Kurki P, Wolff-Holz E, Bielsky MC, Schneider CK (2014). Biosimilars: the science of extrapolation. Blood.

[17] Ebbers HC (2014). Biosimilars: in support of extrapolation of indications. J Crohns Colitis.

[18] Yin D, BarkerK B, Li R (2014). A randomized phase 1 pharmacokinetic trial comparing the potential biosimilar PF-05280014 with trastuzumab in healthy volunteers (REFLECTIONSB327-01). Br J Clin Pharmacol.

